# Supplementary material for: Hepatic macrophages play critical roles in the establishment and growth of hydatid cysts in the liver during Echinococcus granulosus sensu stricto infection
Source: PLoS Negl Trop Dis. 2023 Nov 6;17(11):e0011746. doi: 10.1371/journal.pntd.0011746 (PMC10653610; doi:10.1371/journal.pntd.0011746)
Supplement: S1 Table — (DOCX) [file pntd.0011746.s002.docx]

Table S1 Demographical and clinical characteristics of the CE patients

| **Patient No.** | **Age**  **(years)** | **Gender（F/M）** | **CE Stage** | **ALT (U/L)** | **AST (U/L)** | **ALP (U/L)** | **Characterization of cysts [location, diameter (cm)]** | **Whether or recurrence** |
| --- | --- | --- | --- | --- | --- | --- | --- | --- |
| 1 | 28 | F | 1 | 29.4 | 27.1 | 78.4 | LL, 7.9×6.4 | N |
| 2 | 45 | M | 1 | 114.7 | 102.9 | 127.2 | RL, 7.0×10.0; 6.0×7.0 | Y |
| 3 | 14 | F | 1 | 51.5 | 35.9 | 109.4 | RL, 8.8×8.3;  LL, 10.9×6.6 | N |
| 4 | 55 | F | 1 | 86.0 | 111.0 | 78.0 | RL, 13.4×10.3;  RL, 4.0×4.0 | N |
| 5 | 4 | F | 1 | 79.9 | 49.8 | 279.3 | RL, 9.8×8.8 | N |
| 6 | 8 | F | 1 | 6.5 | 30.2 | 178.3 | RL, 4.3×3.5; 1.5×1.3 | N |
| 7 | 20 | F | 1 | 16.0 | 22.8 | 186.0 | RL, 7.6×3.7; 1.7×1.4 | N |
| 8 | 44 | F | 1 | 28.2 | 26.01 | 93.6 | RL, 11.8×9.3 | N |
| 9 | 48 | F | 1 | 103.0 | 38.0 | 66.0 | RL, 6.0×6.0; 3.0×2.0 | Y |
| 10 | 13 | F | 1 | 172.0 | 124.0 | 61.0 | RL, 8.0×7.0; 2.0×2.0 | N |
| 11 | 50 | M | 1 | 39.9 | 29.0 | 71.7 | RL, 7.0×6.0 | N |
| 12 | 59 | F | 1 | 18.5 | 18.1 | 75.0 | RL, 9.0×7.0; 2.0×1.0 | N |
| 13 | 36 | F | 1 | 18.1 | 19.7 | 68.0 | LL, 8.8×9.0 | N |
| 14 | 43 | F | 2 | 191.6 | 353.6 | 121.6 | RL, 15.3×11.7 | N |
| 15 | 25 | M | 2 | 14.9 | 19.3 | 102.8 | RL, 8.0×6.8;  LL, 6.5×3.3 | Y |
| 16 | 52 | F | 2 | 21.3 | 20.8 | 51.6 | RL, 12.8×8.5 | N |
| 17 | 55 | M | 2 | 17.5 | 15.1 | 108.0 | RL, 9.5×7.1 | Y |
| 18 | 46 | F | 2 | 12.0 | 19.0 | 108.0 | RL, 1.1×1.6;  LL, 13.7×8.0 | N |
| 19 | 27 | F | 2 | 15.8 | 26.8 | 52.2 | RL, 3.8×4.4 | Y |
| 20 | 49 | F | 2 | 97.43 | 71.6 | 139.5 | RL, 10.9×10.0 | Y |
| 21 | 63 | M | 2 | 16.0 | 24.3 | 56.7 | LL, 6.8×6.0 | N |
| 22 | 21 | M | 2 | 35.0 | 30.7 | 97.1 | RL, 17.1×11.5 | N |
| 23 | 72 | M | 2 | 122.1 | 45.1 | 261.5 | RL, 7.7×4.1 | N |
| 24 | 42 | M | 2 | 40.4 | 23.0 | 42.0 | RL, 9.1×8.8 | Y |
| 25 | 62 | M | 2 | 21.9 | 12.4 | 72.0 | RL, 5.37×3.42 | Y |
| 26 | 30 | F | 2 | 20.9 | 22.7 | 55.3 | RL, 9.1×8.1 | Y |
| 27 | 47 | M | 2 | 29.5 | 23.9 | 81.5 | LL, 12.5×12.5 | N |
| 28 | 32 | M | 2 | 32.2 | 63.5 | 132.0 | RL, 5.2×5.8;  LL, 12.4×6.7 | N |
| 29 | 53 | M | 2 | 13.2 | 17.6 | 87.9 | RL, 7.0×5.8; 8.4×5.8 | N |
| 30 | 55 | M | 2 | 18.8 | 18.1 | 104.0 | RL, 8.7×8.4 | Y |
| 31 | 57 | F | 2 | 132.8 | 60.0 | 489.8 | RL, 12.2×9.5 | Y |
| 32 | 29 | M | 2 | 34.9 | 34.7 | 255.0 | RL, 22.1×12.2 | Y |
| 33 | 68 | F | 4 | 12.2 | 17.5 | 100.0 | RL, 5.2×4.8 | N |
| 34 | 48 | F | 4 | 18.7 | 22.6 | 82.4 | RL, 5.3×5.8 | N |
| 35 | 50 | M | 4 | 32.8 | 55.0 | 53.7 | RL, 3.8×3.8; 3.7×3.8 | N |
| 36 | 43 | F | 4 | 22.8 | 17.3 | 43.1 | RL, 4.3×4.1 | N |
| 37 | 53 | M | 4 | 13.2 | 17.6 | 87.9 | RL, 7.0×5.8; 8.4×5.8 | N |
| 38 | 16 | F | 4 | 89.5 | 57.2 | 135.9 | RL, 7.0×6.1 | N |
| 39 | 47 | M | 4 | 17.9 | 19.2 | 58.2 | LL, 6.0×5.1 | N |
| 40 | 57 | F | 4 | 28.4 | 16.8 | 87.0 | LL, 5.9×4.9 | N |
| 41 | 42 | M | 4 | 19.2 | 31.4 | 70.6 | RL, 16.9×13.6 | N |

**Abbreviations:** F, female; M, male; LL, left liver lobe; RL, right liver lobe; ALT, alanine aminotransferase; AST, aspartate aminotransferase; ALP, alkaline phosphatase; N, none; Y, yes.
