# Supplementary figures and images for: Hepatic macrophages play critical roles in the establishment and growth of hydatid cysts in the liver during Echinococcus granulosus sensu stricto infection
Source: PLoS Negl Trop Dis. 2023 Nov 6;17(11):e0011746. doi: 10.1371/journal.pntd.0011746 (PMC10653610; doi:10.1371/journal.pntd.0011746)

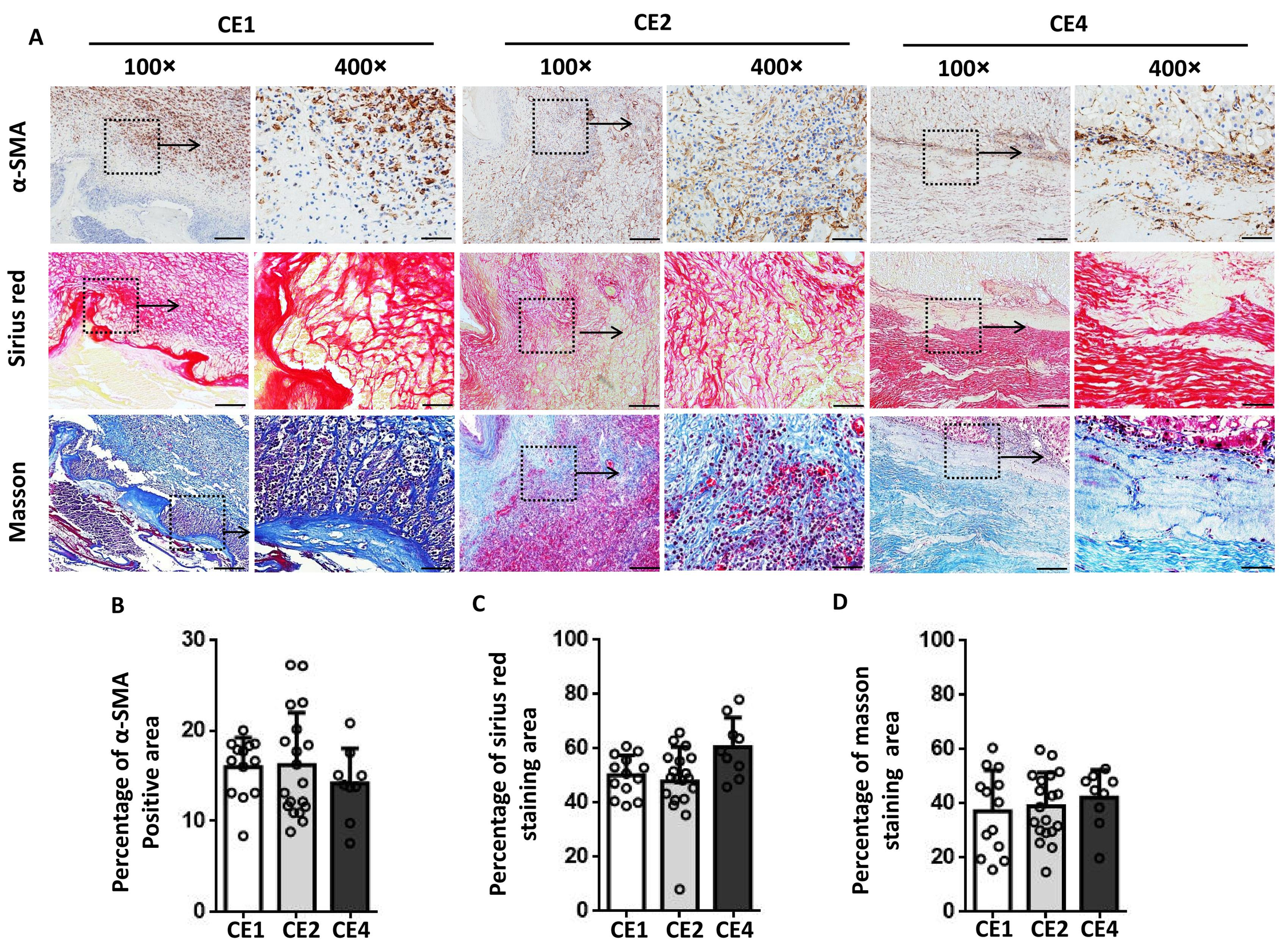

Supplement: S1 Fig — (A) Representative degrees of liver fibrosis, as determined by α-SMA, SR and Masson staining, in liver tissue sections from CE patients with different cyst activity stages (100× and 400× indicate the magnification of the figures; the bars represent 200 and 50 μm for 100× and 400×, respectively). (B-D) The percentages of positive α-SMA, SR and Masson staining were quantified using cellSens Dimension software. The results are presented as the means ± SEMs (n = 13, 19 and 9 for stages CE1, CE2 and CE4, respectively). (TIF) [file pntd.0011746.s003.tif]

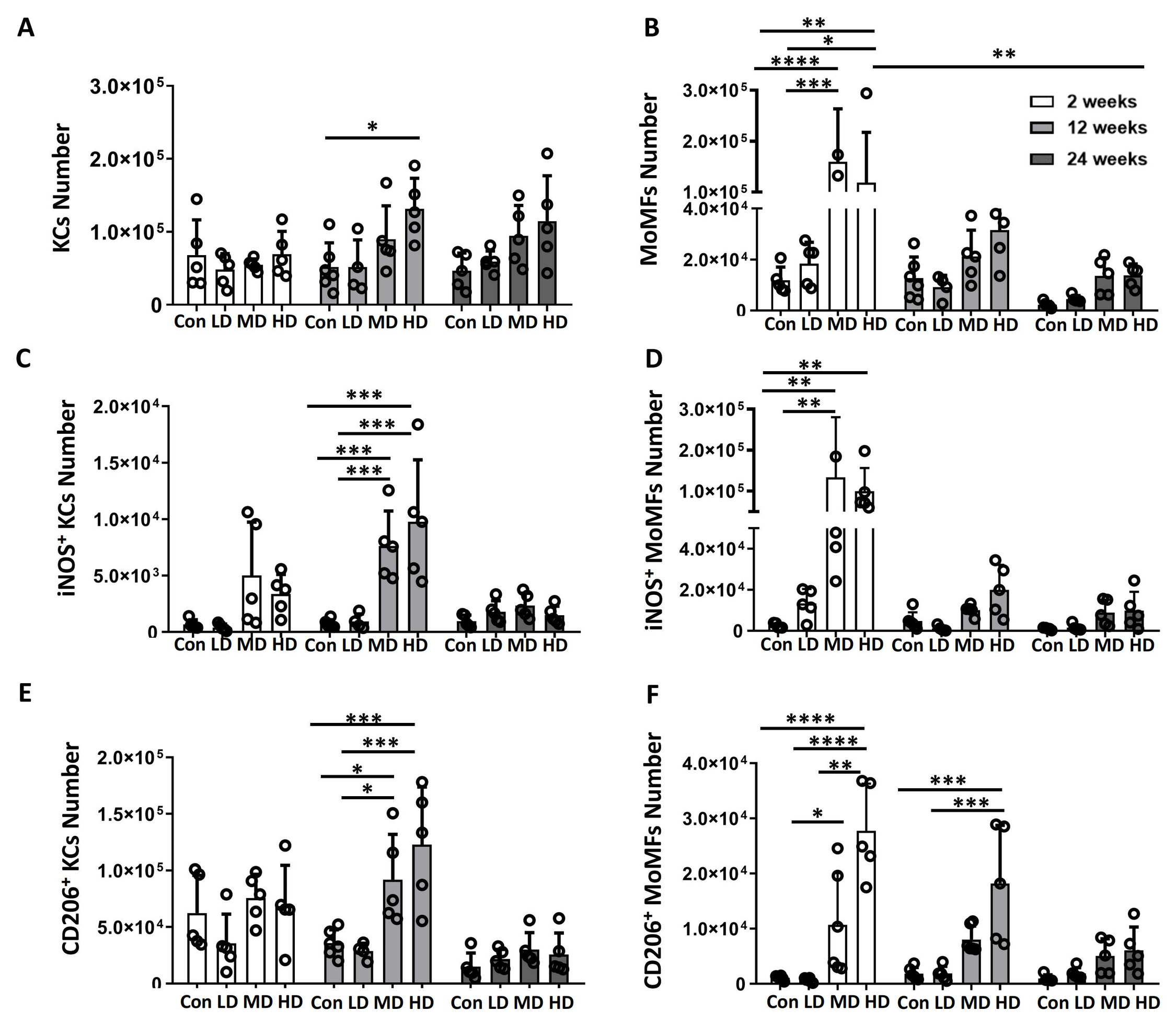

Supplement: S2 Fig — (A, B) Absolute numbers of KCs and MoMFs among the NPLCs of mice infected with different PSC inoculum doses at 2, 12 and 24 weeks after infection. (C, D) Absolute numbers of iNOS+ KCs and iNOS+ MoMFs among the NPLCs of mice infected with different PSC inoculum doses at 2, 12 and 24 weeks after infection. (E, F) Absolute numbers of CD206+ KCs and CD206+ MoMFs among the NPLCs of mice infected with different PSC inoculum doses at 2, 12 and 24 weeks after infection. Con; LD: 50 PSCs; MD: 500 PSCs; HD: 2000 PSCs; n = 5–6 mice per group. The data are shown as the means ± SEMs, *P < 0.05, **P < 0.01, ***P < 0.001 and ****P < 0.0001. (TIF) [file pntd.0011746.s004.tif]

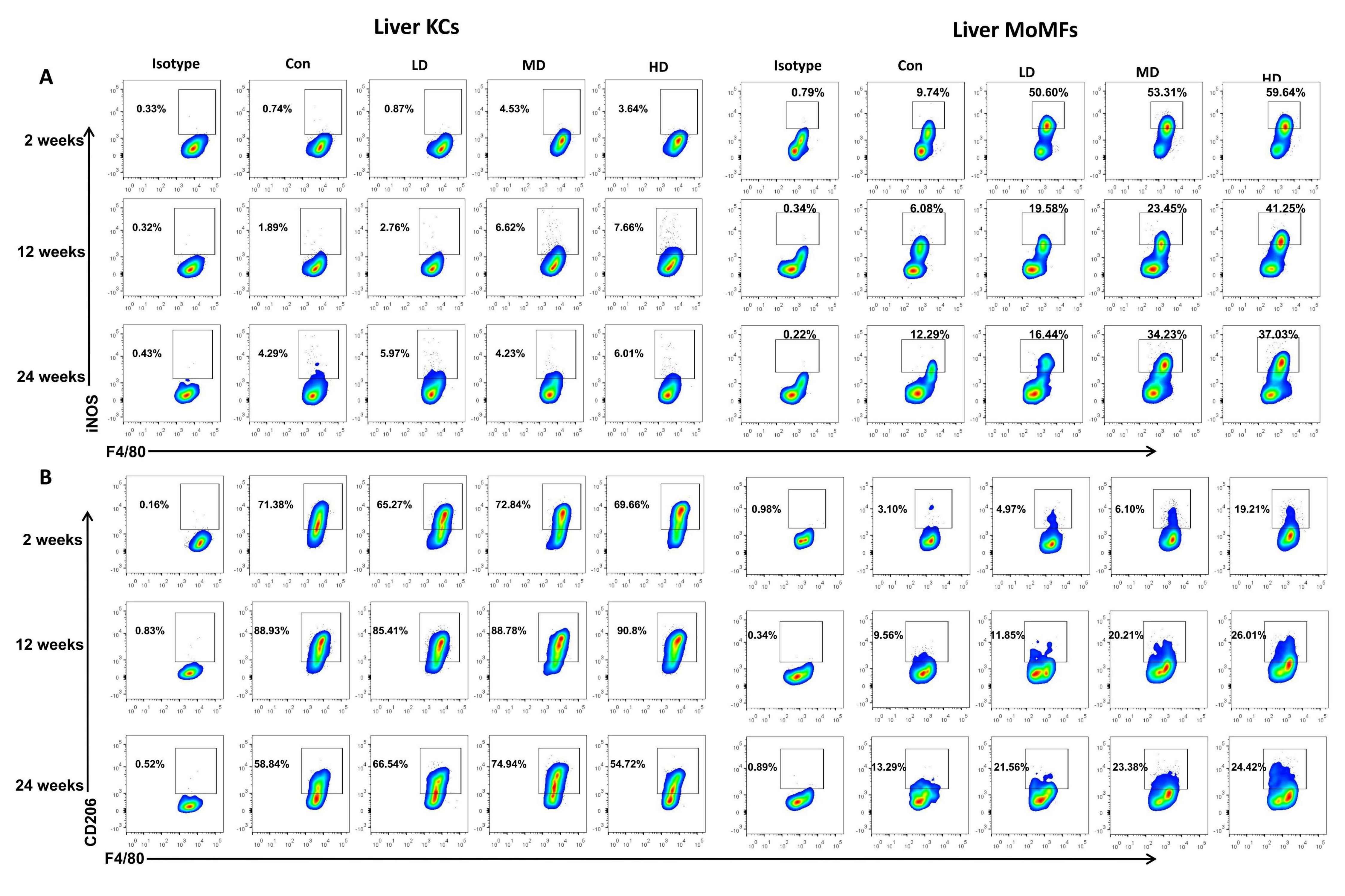

Supplement: S3 Fig — (A) Intracellular staining of iNOS in KCs and MoMFs in the livers of mice infected with different PSC inoculum doses at 2, 12 and 24 weeks post infection. (B) Intracellular staining of CD206 KCs and MoMFs in the livers of mice infected with different PSC inoculum doses at 2, 12 and 24 weeks post infection. Con; LD: 50 PSCs; MD: 500 PSCs; HD: 2000 PSCs. (TIF) [file pntd.0011746.s005.tif]

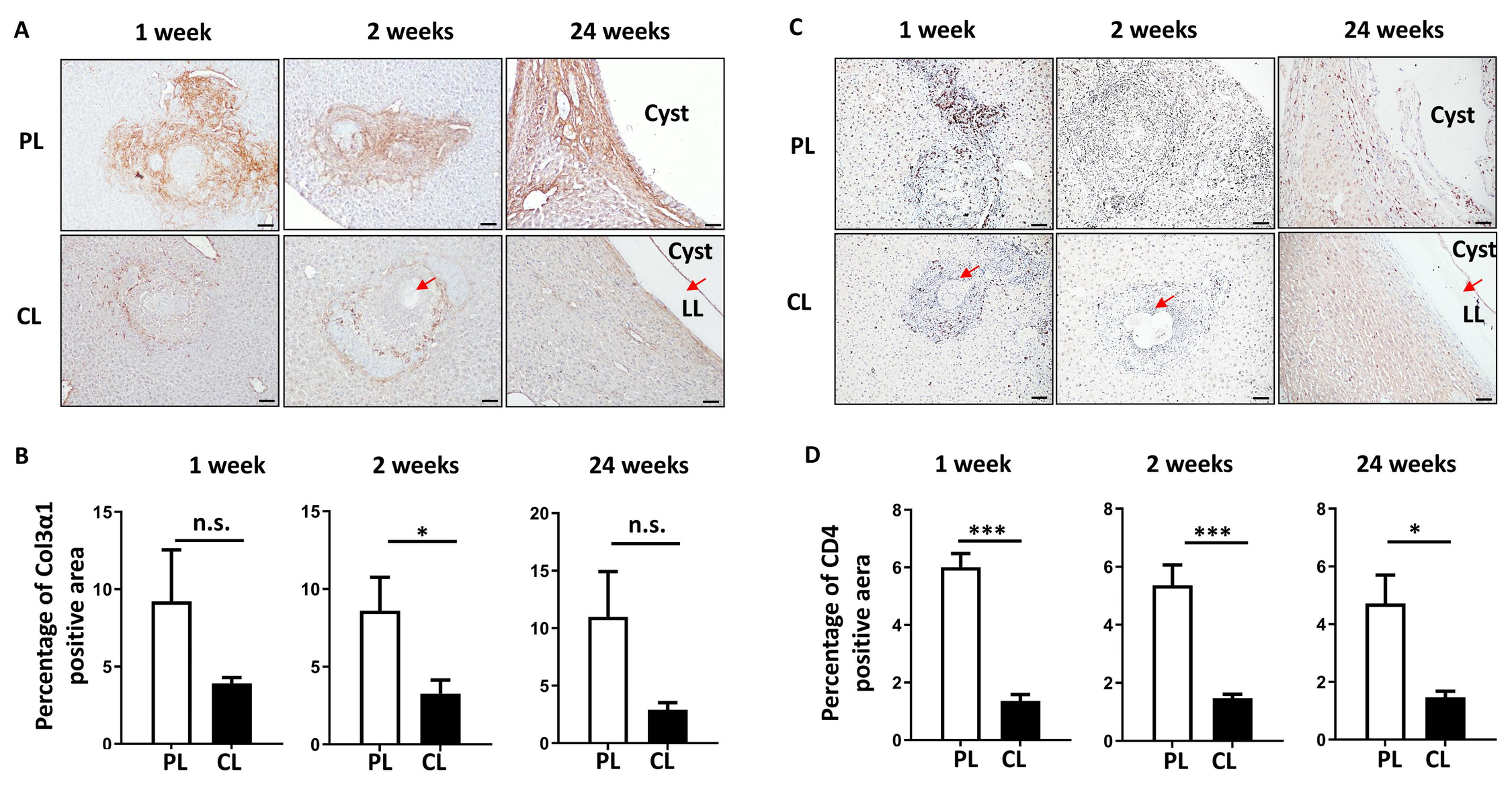

Supplement: S4 Fig — (A) Representative immunohistochemical staining of Col3α1 in liver sections from mice treated with PL or CL at 1, 2 and 24 weeks after inoculation (200× magnification; scale bar, 100 μm). (B) The percentages of positive Col3α1 staining in the liver sections. (C) Representative immunohistochemical staining of CD4 in liver sections from mice treated with PL or CL at 1, 2 and 24 weeks after inoculation (200× magnification; scale bar, 100 μm). (D) The percentages of positive CD4 staining in the liver sections. The red arrows indicate the larvae and LL structure. LL: laminated layer. CL: clodronate liposomes. PL: phosphate-buffered saline control liposomes. The data are shown as the means ± SEMs (n = 4–5 mice per group). *P < 0.05, **P < 0.01 and ***P < 0.001. (TIF) [file pntd.0011746.s006.tif]
