# Supplementary material for: Hepatic macrophages play critical roles in the establishment and growth of hydatid cysts in the liver during Echinococcus granulosus sensu stricto infection
Source: PLoS Negl Trop Dis. 2023 Nov 6;17(11):e0011746. doi: 10.1371/journal.pntd.0011746 (PMC10653610; doi:10.1371/journal.pntd.0011746)
Supplement: Attachment — Submitted filename: comments.docx [file pntd.0011746.s007.docx]

In this manuscript, the authors summarized and discussed the role of hepatic macrophages in establishment and growth of hydatid cysts during *Echinococcus granulosus* infection via infected mouse model. This is a well-conducted study with important results. The manuscript is well-organized and clearly stated. I would suggest accepting it after the following minor concerns are addressed.

1. Please note that different stages of cysts can only represent different hydatid cysts viability, and cannot indicate that the patients are in the early or late stages of infection, as the author wrote in the Author summary, the larvae mainly dwell in the liver and has a long latent period of up to 5 to 10 years.

2. Line439, “in patients with inactive CE4 cysts, macrophages exhibit a dominant proinflammatory S100A9+ M1 phenotype”, however, from Fig.1 F, the percentage of S100A9+ were not significantly different in CE patients at different stages. Please provide more explanation.

3. Fig.5D, what does the Y-axis refer to? If the Y-axis refers to the number of PSCs, does it indicate that the PL group has almost no PSCs in 2 weeks after inoculation? Please provide more details of how to determine the number of PSCs in livers in the Materials and methods.

4. Fig.5 shows that early hepatic macrophage depletion leads to a significant difference in the number of PSCs between PL and CL group, but the late infection of these 2 groups are not seen in the manuscript. If possible, it would be useful if the authors could provide more evidence to demonstrate whether early hepatic macrophage depletion leads to differences in the number and size of formed hydatid cysts.

5. Line 175, what was basis for choosing this time(early stage)?

6. In order to elucidate the role in the progression of CE for the hepatic macrophages, Flow Cytometry, Histopathological and Immunohistochemical Analysis are not enough. Western blot should be added.

7. In the paragraph 4 of results, Why no results of CD68 and CD163? The reason should be discussed in the part of discussion.

8. In the paragraph 2 and 4 of results, the liver fibrosis evaluated by α-SMA positive cells or SR. Why did no western blot test for α-SMA, Col 1 α1, and Col 3 α? And the reason should be discussed.
