# Supplementary material for: Hepatic macrophages play critical roles in the establishment and growth of hydatid cysts in the liver during Echinococcus granulosus sensu stricto infection
Source: PLoS Negl Trop Dis. 2023 Nov 6;17(11):e0011746. doi: 10.1371/journal.pntd.0011746 (PMC10653610; doi:10.1371/journal.pntd.0011746)
Supplement: Attachment — Submitted filename: renamed_98f34.doc [file pntd.0011746.s008.doc]

**Dear Editor and reviewers,**

Thank you very much for providing us with the chance to revise our manuscript entitled “Hepatic macrophages play critical roles in the establishment and growth of hydatid cysts in the liver during *Echinococcus granulosus* sensu stricto infection” (Manuscript ID: PNTD-D-23-00771). We also thank the reviewers for their efforts with providing insightful comments. In this new version of our MS, we have made revisions considering all comments, performed additional experiments and provided more convincing evidence, which specifically addresses the issues raised by the reviewers. We believe that all concerns have been addressed in this revised paper and that the quality of the article has been substantially increased. Below are our point-by-point responses detailing how the manuscript has been revised.

We are now submitting the revised manuscript for your reconsideration for publication in ***PLOS Neglected Tropical Diseases***. Please find two versions of the revised manuscript: one with tracked changes; the other, a clean version (uploaded as the manuscript file). Also, please find our attached responses to the reviewers’ comments.

If any other information is required, please feel free to contact us.

With my best regards,

Sincerely yours,

**Response to the Editor’s comments**

The reviewers have pointed out several key elements of the manuscript that require attention, including but not limited to the results of the analysis of human infections. In addition to the comments of the reviewers, please also include a discussion of the limitations of the murine model, as it is a secondary infection model (using protoscoleces), and the early time points may not be representative of primary infections produced by infective eggs (as occurs in human patients).

**Response:** Thank you for the suggestion. We have revised our MS to address all the reviewers’ comments, and we have included a discussion of the murine model in our revised MS.

Humans accidentally ingest the eggs of *E. granulosus* sensu lato, which hatch in the intestine to release oncospheres that pass through the portal and lymphatic vessels and reach the liver, where they usually settle and develop as larvae (hydatid cysts), causing cystic echinococcosis (CE) ^[1]^. It is very difficult to study the local immune response before and at the early stages of cyst formation because there is a long latent period before CE is diagnosed. Primary infection models are particularly adapted to study host-parasite interactions. However, not only do these models pose a risk for the operator to come in contact with the infectious eggs, the route of infection involves numerous host-dependent steps, and the outcome is dependent on nonimmunological events, such as the presence of gastric and enteric enzymes, bile composition, and the nature of the intestinal barrier. In humans, secondary CE is caused by dissemination of protoscoleces (PSCs) after accidental rupture of fertile cysts and is due to the ability of PSCs to develop into new cysts. This is the reason that the host-parasite immunological relationship has usually been experimentally investigated using a secondary infection model established by direct injection of PSCs, such as a model of intraperitoneal infection ^[2]^. Intraperitoneal infection is widely used because it is a relatively easy and safe technique, but this model does not reproduce the ‘natural’ site of initial parasite development e (i.e., the liver). In our previous studies, we developed a suitable experimental mouse model that can mimic natural liver infection by direct injection of precise numbers of PSCs via the portal vein ^[3,4]^. This model is good for studying early immune responses at the local site of infection establishment in the liver and enables us to study the dynamic changes in hepatic macrophage phenotypes and functions during *E. granulosus* *s.s.* infection.

In addition, we strongly hope that our answers below will address all the reviewer’s concerns and that our modifications will improve the quality of the paper and allow it to be published as soon as possible.

**References**

[1] Wen H, Vuitton L, Tuxun T, Li J, Vuitton DA, Zhang W, et al. Echinococcosis: Advances in the 21st Century. Clin Microbiol Rev. 2019;32(2):e00075-18. doi: 10.1128/CMR.00075-18. PubMed PMID: 30760475.

[2] Mourglia-Ettlin G, Marqués JM, Chabalgoity JA, Dematteis S. Early peritoneal immune response during Echinococcus granulosus establishment displays a biphasic behavior. PLoS Negl Trop Dis. 2011;5(8):e1293. doi: 10.1371/journal.pntd.0001293. Epub 2011 Aug 30. PubMed PMID: 21912714.

[3] Li Z, Zhang C, Li L, Bi X, Li L, Yang S, et al. The local immune response during Echinococcus granulosus growth in a quantitative hepatic experimental model. Sci Rep. 2019;9(1):19612. doi: 10.1038/s41598-019-56098-3. PubMed PMID: 31873157.

[4] Zhang C, Shao Y, Yang S, Bi X, Li L, Wang H, et al. T-cell tolerance and exhaustion in the clearance of Echinococcus multilocularis: role of inoculum size in a quantitative hepatic experimental model. Sci Rep. 2017;7(1):11153. doi: 10.1038/s41598-017-11703-1. PubMed PMID: 28894272.

**Responses to the reviewers’ comments**

**Reviewer #1:**

In this manuscript, the authors summarized and discussed the role of hepatic macrophages in establishment and growth of hydatid cysts during *Echinococcus granulosus* infection via infected mouse model. This is a well-conducted study with important results. The manuscript is well-organized and clearly stated. I would suggest accepting it after the following minor concerns are addressed.

1. Please note that different stages of cysts can only represent different hydatid cysts viability, and cannot indicate that the patients are in the early or late stages of infection, as the author wrote in the Author summary, the larvae mainly dwell in the liver and has a long latent period of up to 5 to 10 years.

**Response:** Thank you for the suggestion. We have noted and modified the corresponding description in the revised MS. The descriptions of early and late stages of infection were only used in the mouse model of *E. granulosus s.s.* infection.

2. Line439, “in patients with inactive CE4 cysts, macrophages exhibit a dominant proinflammatory S100A9+ M1 phenotype”, however, from Fig.1 F, the percentage of S100A9+ were not significantly different in CE patients at different stages. Please provide more explanation.

**Response:** Yes, as seen in Fig. 1G, the percentage of the S100A9-positive area surrounding the cysts was not significantly different in CE patients with cysts at different stages, but the percentage of the S100A9-positive area (M1 macrophages) was significantly higher than that of the CD163^-^positive area (M2 macrophages) in the inactive CE4 specimens (*Please see the following Fig 1*). Thus, we explained that in patients with inactive CE4 cysts, macrophages exhibit a predominant proinflammatory S100A9^+^ M1 phenotype, which may limit hydatid cyst viability in the liver.

Fig. 1 Comparison of the percentage of the S100A9-positive area to the CD163-positive area in samples from CE patients with cysts at different stages.

3. Fig.5D, what does the Y-axis refer to? If the Y-axis refers to the number of PSCs, does it indicate that the PL group has almost no PSCs in 2 weeks after inoculation? Please provide more details of how to determine the number of PSCs in livers in the Materials and methods.

**Response:** In Fig. 5D**,** the Y-axis shows the number of PSCs at 1 and 2 weeks after PSC inoculation. H&E staining of liver sections (Fig. 2B) showed that many PSCs were present and maintained an intact structure in livers from *E. granulosus s.s.*-infected mice at 2 days, with only a few infiltrating lymphocytes surrounding the PSC inoculum were observed. From 5 days to 11 days after infection, PSCs were still observed, but the worm body gradually disintegrated and disappeared because numerous inflammatory cells (including a larger number of proinflammatory monocyte-derived macrophages) were gradually recruited to the area surrounding the PSC inoculum, which promoted early larval clearance. Only a few PSCs were able to develop into hydatid cysts, which were observed from 12 weeks to 24 weeks after infection. This is why almost no intact PSCs were observed in the PL group at 2 weeks after inoculation. However, we observed a higher number of infectious foci with PSCs in the CL group than in the PL group at 1 and 2 weeks because CL treatment markedly reduced F4/80^+^ macrophage and inflammatory cell infiltration into the peripheral areas of the infectious foci with PSCs, and this reduction in infiltration is beneficial for PSC survival.

To determine the number of PSCs in livers at 1 and 2 weeks after infection, whole livers were collected and divided into three sections, fixed with 4% (v/v) paraformaldehyde for 48 hr, embedded in paraffin and then made into three FFPE blocks for H&E staining. Please see the following simplified diagram. Only intact PSCs were counted in the three sections. We have supplemented the details of the method used to determine the number of PSCs in livers in the MATERIALS AND METHODS section according to the reviewer’s suggestion.

4. Fig.5 shows that early hepatic macrophage depletion leads to a significant difference in the number of PSCs between PL and CL group, but the late infection of these 2 groups are not seen in the manuscript. If possible, it would be useful if the authors could provide more evidence to demonstrate whether early hepatic macrophage depletion leads to differences in the number and size of formed hydatid cysts.

**Response:** Administration of clodronate liposomes (CL) to mice is currently the most effective and widely used method to deplete macrophages in vivo ^[1]^. Before this study, we conducted a preliminary experiment to evaluate the depletion efficiency of hepatic macrophages with CL and confirmed that in mice injected intraperitoneally (i.p.) with CL, hepatic macrophages are depleted on day 3 and remain depleted for approximately 7 days (*please see Fig 1 below)*. After 7 days, new macrophages gradually replace the depleted population. To maintain macrophage depletion, mice should be administered multiple injections at 7-day intervals. However, we found that mice can only be administered CL for at most 5-6 times (5-6 consecutive weeks); otherwise, they experience weight loss, limping or even death. However, at 5-6 weeks after infection with *E. granulosus s.s.* PSCs, we observed almost no hydatid cysts in the liver in macroscopic views, since the small hydatid cysts (1-4 mm in diameter) can only be observed at 8 weeks after infection (*Please see the following Fig 2, Li Z, et al. Scientific reports. 2019, 9(1):19612*). Therefore, it is difficult for us to evaluate the impact of early hepatic macrophage depletion on the growth of formed hydatid cysts. Instead, we administered CL to deplete hepatic macrophages at 18 weeks after PSC infection and maintained macrophage depletion for 6 weeks to investigate the impact of hepatic macrophage depletion on the number and size of formed hydatid cysts. We found that macrophage depletion resulted in a high burden of hydatid cysts (Fig 5B in text).

Fig 1. Flow cytometric analysis of the efficiency of hepatic macrophage depletion by intraperitoneal (i.p.) administration of CL.

Fig 2. Macroscopic views of livers from mice infected with *E. granulosus s.s.* during the course of infection.

The yellow circles highlight the hydatid cysts formed in the livers (Cited by Li Z, et al. Scientific reports. 2019;9(1):19612)

**References**

[1] van Rooijen N, Sanders A, van den Berg TK. Apoptosis of macrophages induced by liposome-mediated intracellular delivery of clodronate and propamidine. J Immunol Methods. 1996;193(1):93-9. doi: 10.1016/0022-1759(96)00056-7. PubMed PMID: 8690935.

5. Line 175, what was basis for choosing this time (early stage)?

**Response:** To investigate the status of immune responses, the experimental model of secondary infection has been widely used at the early and late stages after *E. granulosus s.s*. infection. The infection time is typically divided into three stages in most studies: an early stage (until 3-4 weeks post-infection), in which the infection is established, and the middle and late stages (more than 4 weeks post-infection), in which the formed cysts gradually grow and are visible macroscopically ^[1,2,3,4]^. However, there is scant information regarding immune responses at the very early stage (2, 5, 8 and 11 days) in the hepatic mouse model of *E. granulosus s.s*. infection. In addition, based on our previous studies in the *E. multilocularis* infection mouse model ^[5,6],^ we chose the early stage (2, 5, 8, 11 days and 2 weeks) and the middle and late stages (12 and 24 weeks) as time points to investigate the heterogeneity and function of hepatic macrophages during parasite infection.

**References**

[1] Li Z, Zhang C, Li L, Bi X, Li L, Yang S, et al. The local immune response during Echinococcus granulosus growth in a quantitative hepatic experimental model. Sci Rep. 2019;9(1):19612. doi: 10.1038/s41598-019-56098-3. PubMed PMID: 31873157.

[2] Mourglia-Ettlin G, Marqués JM, Chabalgoity JA, Dematteis S. Early peritoneal immune response during Echinococcus granulosus establishment displays a biphasic behavior. PLoS Negl Trop Dis. 2011;5(8):e1293. doi: 10.1371/journal.pntd.0001293. Epub 2011 Aug 30. PubMed PMID: 21912714.

[3] Rostami-Rad S, Jafari R, Yousofi Darani H. Th1/Th2-type cytokine profile in C57 black mice inoculated with live Echinococcus granulosus protoscolices. J Infect Public Health. 2018;11(6):834-839. doi: 10.1016/j.jiph.2018.06.007. Epub 2018 Jul 20. PubMed PMID: 30033229.

[4] Pan W, Zhou HJ, Shen YJ, Wang Y, Xu YX, Hu Y, et al. Surveillance on the status of immune cells after Echinnococcus granulosus protoscoleces infection in Balb/c mice. PLoS One. 2013;8(3):e59746. doi: 10.1371/journal.pone.0059746. Epub 2013 Mar 26. PubMed PMID: 23555767.

[5] Wang H, Zhang CS, Fang BB, Hou J, Li WD, Li ZD, et al. Dual Role of Hepatic Macrophages in the Establishment of the Echinococcus multilocularis Metacestode in Mice. Front Immunol. 2021;11:600635. doi: 10.3389/fimmu.2020.600635. PubMed PMID: 33488594.

[6] Zhang C, Wang J, Lü G, Li J, Lu X, Mantion G, et al. Hepatocyte proliferation/growth arrest balance in the liver of mice during E. multilocularis infection: a coordinated 3-stage course. PLoS One. 2012;7(1):e30127. doi: 10.1371/journal.pone.0030127. Epub 2012 Jan 10. PubMed PMID: 22253905.

6. In order to elucidate the role in the progression of CE for the hepatic macrophages, Flow Cytometry, Histopathological and Immunohistochemical Analysis are not enough. Western blot should be added.

**Response:** Flow cytometry, immunohistochemistry and western blot analysis all utilize the principle of antigen-specific antibody binding to study gene expression at the translational level. Flow cytometry is a technique used to characterize cellular and molecular phenotypes of diverse cell populations in a single-cell suspension, and it is now advanced enough to allow simultaneous analysis of more than 30 parameters in a sample, making the method one of the most powerful tools in biomedical research ^[1,2,3]^. Immunohistochemistry (IHC) is a technique that uses antibodies specific for target antigens to analyze the localization and aberrant expression of genes within tissues. It has the advantages of high specificity, high sensitivity, and accurate localization ^[4]^. Histopathological examination is performed by hematoxylin-eosin staining of tissue sections, which can be used to observe pathological changes such as changes in the status of inflammatory cell infiltration at the sites of local host-parasite responses and parasite development. Western blot analysis has also been widely used in the study of gene expression at the translational level, but first requires the extraction of proteins from liver tissues, which contain many parenchymal and nonparenchymal liver cells, and cannot definitively elucidate the status of hepatic macrophages. Western blotting is a quantitative technique but cannot be used to accurately determine localization.

However, in the present study, we mainly focused on studying the characteristics of hepatic macrophage heterogeneity in the local microenvironment. Therefore, we used flow cytometric analysis to identify the composition and phenotype of hepatic macrophages and used histopathological and immunohistochemical analyses to determine the status of macrophage infiltration and liver fibrosis in the peripheral areas of infectious foci during *E. granulosus s.s.* infection. In addition, due to surgical and ethical limitations, we were unable to obtain liver samples of sufficient volume to meet the requirements for these three detection methods simultaneously. Thus, western blot analysis was not conducted in our present study.

**References**

[1] McKinnon KM. Flow Cytometry: An Overview. Curr Protoc Immunol. 2018 ;120:5.1.1-5.1.11. doi: 10.1002/cpim.40. PubMed PMID: 29512141.

[2] Maciorowski Z, Chattopadhyay PK, Jain P. Basic Multicolor Flow Cytometry. Curr Protoc Immunol. 2017 ;117:5.4.1-5.4.38. doi: 10.1002/cpim.26. PubMed PMID: 28369683.

[3] Pockley AG, Foulds GA, Oughton JA, Kerkvliet NI, Multhoff G. Immune Cell Phenotyping Using Flow Cytometry. Curr Protoc Toxicol. 2015;66:18.8.1-18.8.34. doi: 10.1002/0471140856.tx1808s66. PubMed PMID: 26523471.

[4] Leong AS, Leong TY. Standardization in immunohistology. Methods Mol Biol. 2011;724:37-68. doi: 10.1007/978-1-61779-055-3_3. PubMed PMID: 21370005.

7. In the paragraph 4 of results, Why no results of CD68 and CD163? The reason should be discussed in the part of discussion.

**Response:** In previous studies, CD68 was widely used as a pan-macrophage marker to identify hepatic macrophages in humans. However, in mice, macrophages are usually identified by the expression of the pan-macrophage marker F4/80 ^[1]^. In addition, in humans, the macrophage phenotype was further characterized by the expression of S100A9 (a marker indicating proinflammatory M1 macrophages) or CD163 (a marker indicating anti-inflammatory M2 macrophages). In contrast, in mice, the macrophage phenotype was further characterized by the expression of the M1 marker iNOS or the M2 marker mannose receptor CD206 ^[2-4]^. Thus, in the present study, we used the pan-macrophage marker F4/80 and the M2 marker CD206 or M1 marker iNOS to characterize the phenotypic changes in hepatic macrophages of mice during *E. granulosus s.s.* infection.

**References**

[1] Ritz T, Krenkel O, Tacke F. Dynamic plasticity of macrophage functions in diseased liver. Cell Immunol. 2018 ;330:175-182. doi: 10.1016/j.cellimm.2017.12.007. Epub 2017 Dec 20. PubMed PMID: 29454647.

[2] Krenkel O, Puengel T, Govaere O, Abdallah AT, Mossanen JC, Kohlhepp M, et al. Therapeutic inhibition of inflammatory monocyte recruitment reduces steatohepatitis and liver fibrosis. Hepatology. 2018 ;67(4):1270-1283. doi: 10.1002/hep.29544. Epub 2018 Feb 19. PubMed PMID: 28940700.

[3] Bartneck M, Schrammen PL, Möckel D, Govaere O, Liepelt A, Krenkel O, et al. The CCR2+ Macrophage Subset Promotes Pathogenic Angiogenesis for Tumor Vascularization in Fibrotic Livers. Cell Mol Gastroenterol Hepatol. 2019;7(2):371-390. doi: 10.1016/j.jcmgh.2018.10.007. Epub 2018 Oct 18. PubMed PMID: 30704985.

[4] Wan J, Benkdane M, Teixeira-Clerc F, Bonnafous S, Louvet A, Lafdil F, et al. M2 Kupffer cells promote M1 Kupffer cell apoptosis: a protective mechanism against alcoholic and nonalcoholic fatty liver disease. Hepatology. 2014 ;59(1):130-42. doi: 10.1002/hep.26607.Epub 2013 Nov 20. PubMed PMID: 23832548.

8. In the paragraph 2 and 4 of results, the liver fibrosis evaluated by α-SMA positive cells or SR. Why did no western blot test for α-SMA, Col 1 α1, and Col 3 α? And the reason should be discussed.

**Response:** As mentioned above, in the present study, we mainly focused on the process of liver fibrosis occurrence and development in the peripheral areas of infectious foci during *E. granulosus s.s.* infection. Therefore, we used immunohistochemical staining for α-SMA and SR staining of liver tissue sections to evaluate liver fibrosis in the perilesional areas. These two staining methods have also been widely used in many previous studies to assess liver fibrosis ^[1, 2, 3]^. In addition, according to the reviewer’s suggestion, we also performed immunohistochemical staining for Col3α1 in liver tissue sections to further evaluate liver fibrosis. The results showed the same expression pattern revealed by α-SMA and SR staining: liver fibrosis increased around the infectious foci at the early stage and gradually decreased at the late stage, and hepatic macrophage depletion attenuated Col3α1 staining at 1, 2 and 24 weeks (*please see the following Fig 1&2*).

**References**

[1] Krenkel O, Puengel T, Govaere O, Abdallah AT, Mossanen JC, Kohlhepp M, et al. Therapeutic inhibition of inflammatory monocyte recruitment reduces steatohepatitis and liver fibrosis. Hepatology. 2018;67(4):1270-1283. doi: 10.1002/hep.29544. Epub 2018 Feb 19. PubMed PMID: 28940700.

[2] Miura K, Yang L, van Rooijen N, Ohnishi H, Seki E. Hepatic recruitment of macrophages promotes nonalcoholic steatohepatitis through CCR2. Am J Physiol Gastrointest Liver Physiol. 2012;302(11):G1310-21. doi: 10.1152/ajpgi.00365.2011. Epub 2012 Mar 22. PubMed PMID: 22442158.

[3] Habib A, Chokr D, Wan J, Hegde P, Mabire M, Siebert M, et al. Inhibition of monoacylglycerol lipase, an anti-inflammatory and antifibrogenic strategy in the liver. Gut. 2019;68(3):522-532. doi: 10.1136/gutjnl-2018-316137. Epub 2018 Oct 9. PubMed PMID: 30301768.

Fig 1. Immunohistochemical analysis of Col3α1 staining in mouse livers infected with *E. granulosus s.s.* protoscolex (PSC) inocula at different times after infection.

**(A)** Representative immunohistochemical staining of Col3α1 in liver sections from *E. granulosus s.s.*-infected mice (200× indicates the magnification of the figures; the bars represent 100 μm). **(B)** The percentages of positive Col3α1 staining in liver sections from 4-5 mice per time point. The data are shown as the means ±SEMs. **P* < 0.05, ***P* < 0.01, ****P* < 0.001.

Fig 2. Hepatic macrophage depletion reduces Col3α1 accumulation around liver lesions in *E. granulosus s.s.*-infected mice.

**(A)** Representative immunohistochemical staining of Col3α1 in liver sections from mice treated with PL or CL at 1, 2 and 24 weeks after inoculation (200× magnification; scale bar, 100 μm). **(B)** The percentages of positive Col3α1 staining in the liver sections. The red arrows indicate larvae and the LL structure. LL: laminated layer. CL: clodronate liposomes. PL: phosphate-buffered saline control liposomes. The data are shown as the means ± SEMs (n = 4-5 mice per group). **P* < 0.05, ***P* < 0.01 and ****P* < 0.001.

**Reviewer #2:**

In this study, the authors characterize the immune events involved in the establishment and growth of echinococcal cysts focusing on the role of macrophages. The in vivo studies, in which timely evaluation is performed, are properly designed and allow to follow the echinococcal cyst from the infection to its establishment and growth, and therefore to characterize the role of macrophages in the different stages of infection.

In contrast, the characterization of the “history” of echinococcal cysts in humans is a complex topic. Indeed, the current knowledge on the natural history of the cysts rely on mass screening studies by ultrasound; moreover, we need to consider that cysts with morphological aspects suggesting viability may actually be not viable and vice versa. Based on this assumption, the main concern regarding this study is the lack of study population description for the human study. A paragraph in the RESULTS section on the study population is missing. Inclusion and exclusion criteria must be reported; moreover, for each clinical group it is important to know if enrolled patients have only hepatic cysts or have cysts located in both liver and other organs; data on pharmacological treatment prior enrollment are also important. Pharmacological treatment information are crucial for clinical stage definition mainly for CE4 cysts as, CE4 cysts resulting from treated CE3b, even presenting pathognomonic features of inactive cysts, may reactivate overtime. Therefore, CE4 cysts may be considered inactive and not viable only if spontaneously inactivated or if the last treatment goes back several years before enrollment. The authors refer to the WHO-IWGE classification system for CE1-CE5 staging, however, imprecise concepts are reported. Indeed, the classification in active/transitional/inactive clinical groups is reported in ref 19 (WHO Informal Group Acta Trop 2003), whereas ref 20 (Hosch et al 2008) correlates the metabolite profiles of cysts with cyst activity/viability defined by ultrasound. Moreover, Hosch et al reports that CE3a have equal probability of being viable or nonviable, whereas CE3b cysts are usually viable (in the manuscript CE3 are described as CE3a!!!). Finally, a Table reporting all patients demographical and clinical characteristics is needed.

**Response:** Thank you for the comments. We have carefully revised this article according to your suggestions. We have added a detailed table of patient demographical and clinical characteristics, including age (years), sex (F/M), stage of CE, ALT (U/L), AST (U/L), ALP (U/L), cyst characteristics [location, diameter (cm)], and whether or recurrence (Table S1). We have also added a paragraph on the study population in the RESULTS section. Moreover, as the reviewer mentioned, stage CE3 indicates a transitional stage, with an equal probability of viability or nonviability ^[1,2]^. Thus, patients who had CE3 cysts were not included in this study.

**References**

[1] WHO Informal Working Group. International classification of ultrasound images in cystic echinococcosis for application in clinical and field epidemiological settings. Acta Trop. 2003;85(2):253-61. doi: 10.1016/s0001-706x(02)00223-1.PubMed PMID: 12606104.

[2] Almulhim AM, John S. Echinococcus Granulosus. In: StatPearls [Internet]. Treasure Island (FL): StatPearls Publishing; 2023 . PubMed PMID: 30969573.

Minor points:

1.English and editing need to be deeply revised

**Response:** According to the reviewer’s suggestion, we have used Springer Nature Author Services to make thorough revisions to this article. The corresponding certificates are provided as follows.

2.Lines 115-117: Please revise the sentence: the “early” and the “late” reaction to *E.granulosus* in humans is difficult to define.

**Response:** According to the reviewer’s suggestion, we have revised this sentence.

3.Line 206-207: “cells were first….at 4°C and” is redundant

**Response:** According to the reviewer’s suggestion, we have deleted this text.

4. In the MATERIAL and METHODS section the authors stated that 9 patients with CE4 or CE5 cysts were enrolled. However, only data on CE4 patients are reported. Why patients with CE5 were excluded? If CE4-CE5 patients are considered as a single group, this should be clearly stated in the RESULTS section and figures should be modified accordingly. Moreover, please consider the importance of treatment for groups classification.

**Response:** Thank you for the suggestion. In the present study, the group of patients with inactive CE included only 9 patients with CE4 cysts. According to the treatment principles for hepatic cysts ^[1],^ for patients with CE5 cysts with a solid cyst wall, the "watch and wait" approach is usually considered, and surgical treatment is rarely used. Therefore, it is difficult for us to collect liver specimens from patients with CE5 cysts. We considered the importance of treatment for group classification in this study.

**References**

[1] Brunetti E, Kern P, Vuitton DA; Writing Panel for the WHO-IWGE. Expert consensus for the diagnosis and treatment of cystic and alveolar echinococcosis in humans. Acta Trop. 2010;114(1):1-16. doi: 10.1016/j.actatropica.2009.11.001. Epub 2009 Nov 30. PubMed PMID: 19931502.

5. In the flow cytometry paragraph of the MATERIAL and METHODS section: how many cells per group were acquired at 2, 12 and 24 weeks?

**Response:** At 2, 12 and 24 weeks, the whole infected liver of each mouse in each dose group was used to isolate nonparenchymal liver cells (NPLCs), and we separated 1.0×10^6^ NPLCs for flow cytometry with antibody labeling and acquired 2.0×10^5^ cells from each sample on LSRFortessa flow cytometer for data analysis. We have added these details to the flow cytometry subsection of the MATERIALS AND METHODS section in the revised MS.

6. lines 257-262: the results should be described more precisely: CD68 is significantly increased in CE1 compared to CE2 (that is a biologically viable stage) and to CE4. Moreover, CE2 and CE4 have similar percentages of CD68+ cells. Could the author comment these data?

**Response:** We have modified the description of the results as precisely as possible according to the reviewer’s suggestion. The percentages of CD68^+^ total macrophages in patients with active CE2 cysts were not significantly higher than those in patients with inactive CE4 cysts. However, the percentages of CD163^+^ anti-inflammatory M2 macrophages, which may favor hydatid cyst viability in the liver, were significantly higher in patients with active CE2 cysts than in patients with inactive CE4 cysts. Furthermore, we should collect more clinical samples in the future to confirm these findings.

7. What the author means for “absolute number” of KCs or MoMFs? In the MATERIAL and METHODS section only percentage of positive cells is mentioned.

**Response:** The “absolute number” of KCs or MoMFs in this study refers to the number of KCs or MoMFs, respectively, in the whole liver of each mouse. The absolute number of cells was calculated by the following formula: number of nonparenchymal cells in the whole liver × percentage of KCs or MoMFs among the total liver population of nonparenchymal cells. According to the reviewer’s suggestion, we have added the description of the absolute cell number to the MATERIALS AND METHODS section in the revised MS.

8. Lines 318-330: the results should be described more precisely.

**Response:** According to the reviewer’s suggestion, we have added as precise a description as possible in the revised MS.

Figure Files:

While revising your submission, please upload your figure files to the Preflight Analysis and Conversion Engine (PACE) digital diagnostic tool, https://pacev2.apexcovantage.com.PACE helps ensure that figures meet PLOS requirements. To use PACE, you must first register as a user. Then, login and navigate to the UPLOAD tab, where you will find detailed instructions on how to use the tool. If you encounter any issues or have any questions when using PACE, please email us at [figures@plos.org](mailto:figures@plos.org).

**Response:** We have uploaded our figure files to the PACE digital diagnostic tool and confirmed that all figures meet the PLOS requirements.

Data Requirements:

Please note that, as a condition of publication, PLOS' data policy requires that you make available all data used to draw the conclusions outlined in your manuscript. Data must be deposited in an appropriate repository, included within the body of the manuscript, or uploaded as supporting information. This includes all numerical values that were used to generate graphs, histograms etc.. For an example see here: [http://www.plosbiology.org/article/info%3Adoi%2F10.1371%2Fjournal.pbio.1001908#s5](http://www.plosbiology.org/article/info%3Adoi%2F10.1371%2Fjournal.pbio.1001908#s5).

**Response:** According to the PLOS data policy requirements, we have uploaded all raw data as a supporting information file.

**Reproducibility:**

To enhance the reproducibility of your results, we recommend that you deposit your laboratory protocols in protocols.io, where a protocol can be assigned its own identifier (DOI) such that it can be cited independently in the future. Additionally, PLOS ONE offers an option to publish peer-reviewed clinical study protocols. Read more information on sharing protocols at <https://plos.org/protocols?utm_medium=editorialemail&utm_source=authorletters&utm_campaign=protocols>

**Response:** The laboratory protocols used in this study can be directly obtained by contacting the first and corresponding authors.
